# The potential of aging rejuvenation

**DOI:** 10.1080/15384101.2021.2013612

**Published:** 2022-01-03

**Authors:** Ana O’Loghlen

**Affiliations:** Epigenetics & Cellular Senescence Group, Blizard Institute, Barts and the London School of Medicine and Dentistry, Queen Mary University of London, London UK

**Keywords:** Senescence, aging, rejuvenation, extracellular vesicles, intercellular communication, SASP

## Abstract

Aging is a process by which basic cellular functions and tissue homeostasis start to decline and organs become progressively dysfunctional. Although aging was once considered irreversible, the concept of the elixir of youth or rejuvenation has been in the history for centuries. In fact, recent scientific studies now show the existence of alternative strategies to delay aging. Here, we discuss how different signaling pathways, a variety of cell types and molecules can contribute to delay aging. In addition, we will define recently described rejuvenation strategies, with an emphasis on the potential for extracellular vesicles (EV).

The number of elderly people has dramatically increased worldwide in the last decades. As a consequence, diseases associated with aging such as cancer and neurodegenerative diseases are on the rise. However, a number of rejuvenation strategies or strategies that delay aging have been found in the last decades. These target various age-related pathways and include a combination of cells, molecules and vesicles. This review aims to link all strategies in order to gain a better understanding of the potential of extending lifespan.

## Pathways, molecules, and extracellular vesicles can regulate rejuvenation

There are several main pathways that have been described to regulate the aging process. The nutrient sensing pathways AMP-activated protein kinase (AMPK), mammalian target of rapamycin (mTOR) and insulin and insulin-like growth factor (ISS) pathways are some of these pathways. In fact, blocking AMPK, ISS and mTOR have been shown to delay aging in different animal models from worms to mice [1,2]. Complementary to these pathways and also involved in regulating aging are sirtuins. Sirtuins are enzymes that act mainly through their deacetylase activity that is dependent on the levels of nicotinamide adenine dinucleotide (NAD^+^) [3,4]. NAD^+^ is an important cellular cofactor and signaling molecule regulating many biological processes including metabolism, oxidative state and cell survival that declines with aging. Thus, boosting NAD^+^ levels has proven to be efficient in delaying aging in several models of accelerated aging such as progeroid and BubR1 mice. Interestingly, other life-extending strategies such as caloric restriction partially lead to an increase in NAD^+^ and sirtuin activity levels [5]. However, although several clinical trials are in place for testing NAD^+^ boosting drugs and their benefits in human health [4] not all strategies have been successful [5–9]. In fact, NAD^+^-augmenting dietary supplements were found to be protumorigenic in certain scenarios in mice models [10].

Apart from nutrient response molecules and pathways, there are other factors that have been implicated in rejuvenation. The heterochronic parabiosis experiments where the circulatory system of aged and young mice is shared show the presence of both pro-aging factors that negatively influence young mice, and rejuvenation factors that ameliorate aging in several tissues in naturally aged mice [11]. A great effort has been placed to identify unique rejuvenating mediators with some studies pinpointing this role to growth differentiation factor 11 (GDF11), tissue inhibitor of metalloproteinases 2 (TIMP2), Mesencephalic Astrocyte Derived Neurotrophic Factor (MANF) or oxytocin [12–15]. Although the individual administration of some of these factors improves singular tissue function, it is likely that a combination of all, in addition to other unknown factors, mediate overall aging rejuvenation. In fact, recent studies have shown that extracellular vesicles (EV), which are membrane-protected vesicles that contain an assortment of proteins, nucleic acids, lipids, and metabolites can mediate aging rejuvenation [16–18]. EV can be classified byorigin, being either endocytic or forming directly from the plasma membrane o by size, with those ranging between 50 and 150 nm termed small extracellular vesicles (sEV) [19]. It has been shown that treatment of middle-aged mice with EV isolated from hypothalamic neural stem cells (NSC) increased the lifespan of these mice compared to aged-matched controls. The authors showed that these effects were due to microRNA (miR) enrichment in these EV and suggested that NSC were controlling aging partially through these miR-enriched EV [18]. On the other hand, the NAD^+^ biosynthesis enzyme, nicotinamide phosphoribosyltransferase (NAMPT), was found enriched in EV in young mice, while its levels decreased with aging. NAMPT can be found as an intracellular form (iNAMPT), which is the rate limiting enzyme implicated in the formation of NAD^+^, while its extracellular form (eNAMPT) can have cytokine-like functions [5]. Remarkably, intraperitoneal injection of eNAMPT-enriched EV isolated from 4–12 month old mice into 26 month old female mice showed an increase median and maximal lifespan [16] similar to the lifespan increase observed when inhibiting the mTOR pathway by rapamycin [20]. It would be interesting to investigate whether similar results would be found in male mice, as studies have shown sex differences in life-span [21]. Although the authors did not look into mTOR activity, they did confirm induction of the NMN/NAD^+^ biosynthesis pathway in the hypothalamus, hippocampus, pancreas and retina of adipose specific *Nampt* knock-in mice suggesting a systemic role for adipose eNAMPT-enriched EV [16]. Similarly, a recent study from our lab has found amelioration of senescence-related biomarkers in 22–25 month old mice injected with sEV isolated from young fibroblasts. We previously showed by mass spectrometry analysis that sEV protein content was important during senescence [22]. Data reanalysis identified an enrichment in the glutathione-S-transferase μ2 (GSTM2) in sEV from primary young human donor fibroblasts in comparison from sEV from old fibroblasts [17,22]. Importantly, sEV from young donors comprised intrinsic GST activity which was not present in the soluble fraction. Thus, sEV induced GST activity and reduced lipid peroxidation in the liver, brown adipose tissue, kidney and serum of sEV-treated old mice [17]. It is interesting to note that both NAD^+^ and glutathione’s levels decrease with aging [23,24] and that supplementing or enriching these within EV delays aging or senescence-related features to an extent [16,17]. Altogether, these studies show the rejuvenation potential of EV in aging. In spite of each study attributing their beneficial effects on delaying aging to miR, eNAMPT or GSTM2, it is likely that a combination of all together with other EV and non-EV components in addition to added unknown factors are involved.

## Many cells are involved in the rejuvenation process

Most studies pin-point a major role in aging rejuvenation to an increase in stem cell number and their regenerative state [25]. In fact, several rejuvenation studies observe improvements in stem cell performance [2,26]. For example, implantation of genetically modified hypothalamus NSC into middle-aged mice delays aging related features and increases longevity [18]. However, it is likely that other cell types also play an important role in the rejuvenation process. For instance, it has been shown that macrophages are key for the rejuvenation of age-associated decline in remyelination efficiency of oligodendrocyte precursor cells [27], while adipocytes in an adipose specific *Nampt*-knockin female mice induced longer healthspan and increased physical activity through EV [16]. Interestingly, cell-free blood-borne factors and umbilical cord plasma recapitulate some features of the parabiosis experiments [2]. As these fluids are enriched in factors and EV which are released by a variety of cell types such as endothelial cells and blood cells it confirms the importance other cell types, apart from stem cells, during rejuvenation [15,28]. In fact, EV from mesenchymal stem cells (MSC) and fibroblasts also have rejuvenating properties, although the heterogeneity, isolation protocols and biological properties of MSC should be further validated [17,29,30]. Although many studies show a correlation between a decrease in the number of senescent cells or reversibility of the senescence phenotype due to interventions such as reprogramming and/or sEV treatment [17,31–33], whether this is due to reversibility of senescence, rejuvenation of nearby cells or by potentiating the immunosurveillance should be taken into account and further validated [34].

## Strategies that mediate rejuvenation

**Figure 1.**
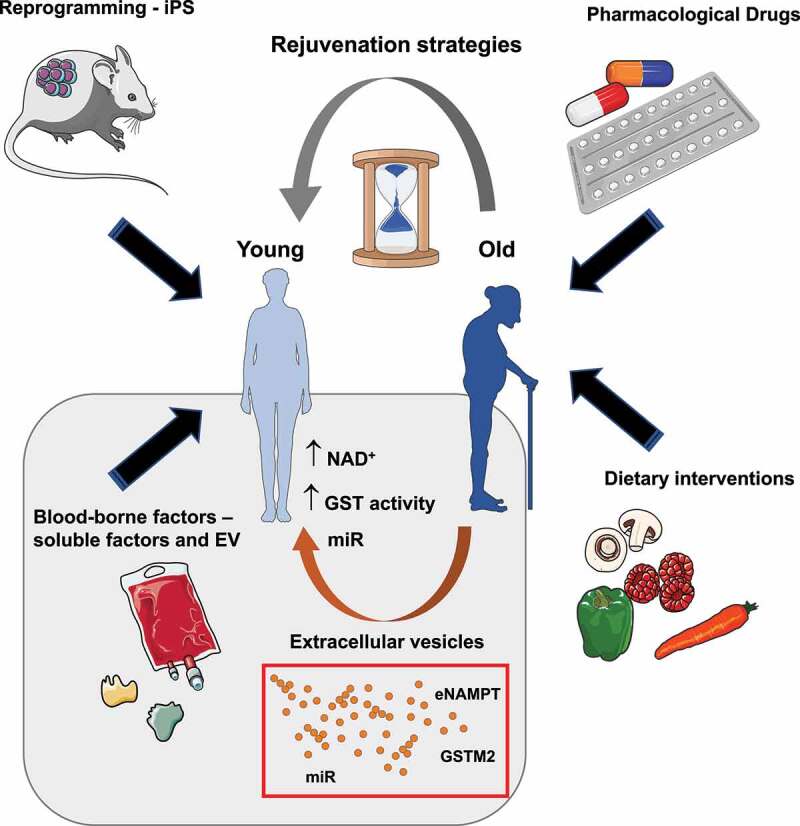
Current rejuvenation strategies to promote healthy **aging**. There are a few strategies that have been proven to improve aging or age-related diseases. Some include the use of pharmacological drugs targeting nutrient sensitive pathways implicated in regulating aging, Other strategies include dietary restrictions, such as low caloric intake or reduced carbohydrate/protein consumption, that affect the nutrient-sensitive pathways. A bit more challenging is the generation and partial induction of induced pluripotent stem cells (iPS) by the expression of the four Yamanaka factors: Oct4, Sox2, Klf4 and c-Myc (OSKM). Finally, the use of cell-free blood-borne factors and in particular extracellular vesicles (EV) is an emerging strategy that holds promise for the clinic once some technical barriers are achieved. In fact, EV enriched in nicotinamide phosphoribosyltransferase (NAMPT), glutathione-S-transferase mu2 (GSTM2) or a specific subset of microRNAs (miR) have been shown to stimulate rejuvenation by promoting the biosynthesis of nicotinamide adenine dinucleotide (NAD^+^) or activating the GST pathway. It is important to note that it is likely all or at least some pathways are interconnected contributing to organismal rejuvenation.

There are currently a variety of strategies to promote healthy aging and/or rejuvenation (Figure 1). Some involve altering the nutrient-related pathways either by dietary restrictions or through pharmacological interventions employing chemical drugs such as rapamycin, metformin or resveratrol [2]. Others involve the use of individual “rejuvenating” factors as MANF, TIMP2, GDF11 [2,26]. Importantly, in the last years the use of drugs eliminating senescent cells – termed senolytics- or dampening their secretome – senomorphics – has caught much attraction in the scientific community [23,35]. In fact, the use of senolytics in different animal models has shown an overall improvement on aging and age-related diseases [35] which could complement one another when used in combination with other anti-aging therapies. Nonetheless, more research is needed on the role of these promising drugs on the overall improvement of an organism, as the presence of senescent cells is important for tissue repair and tumor suppression mechanisms.

Other rejuvenation strategies involve the temporary induction of pluripotent stem cells (iPS) [24]. Interestingly, partial induction of iPS by expression of the four Yamanaka factors (Oct4, Sox2, Klf4 and c-Myc: OSKM) induces senescence both *in vivo* and *in vitro* [36,37]. The senescence-associated secretory phenotype (SASP) from these iPS-senescent cells induce *in vivo* reprogramming and cellular plasticity in aging and progeroid animal models, mainly through interlukin-6 (IL-6) [31,32,38]. This plasticity property is similar to what is observed with sEV from young donors [16–18] and from sEV isolated from iPS cells (unpublished data from our lab). However, whether there are common features between the EV released by these different cell types or whether they affect similar or different pathways to the SASP released by iPS is unknown.

EV are found as cell-free blood-borne components and are an alternative rejuvenation strategy that remains largely unexplored. EV contain proteins, lipids, nucleic acids, and metabolites that are protected by a lipid membrane making them more resistant to circulating proteases than free protein. Importantly, it has been recently shown by different groups that EV hold intrinsic metabolic activity making them traveling “metabolic” units with the capability of changing the metabolomic profile of the tissues incorporating these EV [16,17]. Furthermore, they do not induce toxicity nor activation of the immune system as they are immunogenic. The fact that they are cell-free also makes them safer than cell-transplant strategies. In spite of all this, the heterogeneity of EV released by single cells and the lack of a high throughput isolation protocols hamper the use of EV as rejuvenation therapies for now.

## Future perspectives

It is exciting to note that different strategies mediate similar rejuvenation effects. However, this can also be considered a shortcoming in our understanding of the rejuvenation process. The fact that several strategies and a variety of pathways, molecules, and cell types are involved suggest a much more complex situation that the one presented so far. Thus, the interconnection between all these factors is also unknow and it is possible that a combination of strategies targeting a multitude of pathways is required for true organismal rejuvenation. Furthermore, the knowledge of whether all these processes are truly inducing whole-organism rejuvenation or promoting a transient single-tissue improvement is unclear. Finally, several studies have proven tissue-specific rejuvenation, suggesting that each different tissue might need an individual and very specific rejuvenation strategy. In spite of this and although more research is needed, all these studies show the potential of promoting healthy aging across lifespan.
